# Vitamin B_12_ Enhances Nerve Repair and Improves Functional Recovery After Traumatic Brain Injury by Inhibiting ER Stress-Induced Neuron Injury

**DOI:** 10.3389/fphar.2019.00406

**Published:** 2019-04-24

**Authors:** Fangfang Wu, Ke Xu, Lei Liu, Kairui Zhang, Leilei Xia, Man Zhang, Chenhuai Teng, Heyan Tong, Yifang He, Yujie Xue, Hongyu Zhang, Daqing Chen, Aiping Hu

**Affiliations:** ^1^Department of Emergency, The Second Affiliated Hospital and Yuying Children’s Hospital, Wenzhou Medical University, Wenzhou, China; ^2^School of Pharmaceutical Sciences, Wenzhou Medical University, Wenzhou, China; ^3^Institute of Life Sciences, Wenzhou University, Wenzhou, China; ^4^Department of Emergency, Wenzhou People’s Hospital, The Third Clinical Institute Affiliated to Wenzhou Medical University, Wenzhou, China

**Keywords:** vitamin B_12_, traumatic brain injury, endoplasmic reticulum stress, microtubule, myelin

## Abstract

Traumatic brain injury (TBI) is one of the most common causes of neurological damage in young human populations. Vitamin B_12_ has been reported to promote axon growth of neuronal cells after peripheral nerve injury, which is currently used for the treatment of peripheral nerve damage in the clinical trial. Thus, we hypothesized that TBI can be attenuated by vitaminB_12_ treatment through its beneficial role on axon regeneration after nerve injury. To confirm it, the biological function of vitaminB_12_ was characterized using hematoxylin and eosin (H&E) staining, Luxol fast blue (LFB) staining, western blot analysis, and immunohistochemistry staining. The results showed that the neurological functional recovery was improved in the VitaminB_12_-treated group after TBI, which may be due to downregulation of the endoplasmic reticulum stress-related apoptosis signaling pathway. Moreover, the microtubule stabilization, remyelination and myelin reparation were rescued by vitamin B_12_, which was consistent with the treatment of 4-phenylbutyric acid (4-PBA), an endoplasmic reticulum stress inhibitor. The study suggests that vitamin B_12_ may be useful as a novel neuroprotective drug for TBI.

## Introductions

Traumatic brain injury (TBI) is commonly found following traffic accidents in adults or perinatal asphyxia in newborns, which causes brain swelling with an increase in intracranial pressure and a subsequent decrease in cerebral perfusion leading to ischaemia (Fink et al., 2012). Previous therapeutic approaches have focused on protecting the blood-brain-barrier at the early stage of injury to mitigate damages such as ionic homeostasis disturbances, secondary cerebral oedema, inflammation and the generation of free radicals (Mayer et al., 2013; Katz et al., 2015; Ma et al., 2017). The repair processes following TBI are severely limited due to a failure to entirely replenish the neuronal population (Sun, 2014). Moreover, the degeneration and necrosis of axons are also the pivotal pathological event of acute TBI (Zhang et al., 2015). Therefore, inhibiting neuronal apoptosis and promoting axon regeneration are of great significance in promoting the functional recovery from TBI (Sun, 2014; Zhang et al., 2016).

Microtubules stability is a major determinant of axonal growth and neuronal polarization during axon formation (Murillo and Sousa, 2018). A previous study had demonstrated that the administration of fibroblast growth factor 13 (FGF13) maintained microtubule stability, and promoted axon formation and neuronal polarization after spinal cord injury (Li et al., 2018). Microtubule-stabilizing proteins (MSPs), as cytoskeletal proteins, cross cells and transport nutrients to ensure the integrity of cell function (Nogales, 2000). Members of the MSP family, such as microtubule-associated proteins (MAPs), Tau and doublecortin (DCX) can promote microtubule assembly, stabilize the microtubule, and play key roles in directing neuronal migration into the cerebral cortex and axon regeneration at the early stage of neuronal migration (Takei et al., 2000; Kapitein and Hoogenraad, 2015; Brunden et al., 2017). Thus, a drug that reduces neuronal apoptosis and promotes microtubule stability may represent a promising approach for the clinical treatment of TBI (Brunden et al., 2017; Gao et al., 2018).

Vitamin B_12_ (Mecobalamin, MeCbl) is an important micronutrient that is required in numerous biological processes (Rathod et al., 2016). It is considered a coenzyme in folate metabolism and nucleotide biosynthesis, which makes it crucial in the metabolism of fatty acids and some amino acids and normal nervous system function (Field et al., 2018). Furthermore, vitamin B_12_ deficiency results in methionine deficiency, leading to the dyes-synthesis of both phospholipids and myelin (Gröber et al., 2013). Currently, combination therapy with vitamin B_12_ is widely combined and used in clinical patients with nerve diseases. It has been reported that systemic administration of vitamin B_12_ promoted the recovery process from peripheral nerve damage in experimental rats (Hobbenaghi et al., 2013). Additionally, vitamin B_12_ was recently shown to be a superoxide scavenger contributing to neuronal cells axonal growth (Chan et al., 2018). Thus, we hypothesized that vitamin B_12_ could enhance axon formation after TBI via stabling microtubule and reducing neuronal apoptosis.

The accumulation of misfolded protein in the endoplasmic reticulum (ER) leads to ER dysfunction, which is known as ER stress (Hughes and Mallucci, 2018). Recent studies have demonstrated that ER stress is involved in a range of neurological diseases, including cerebral ischaemia, neuro-degenerative disorders and Alzheimer’s disease (Roussel et al., 2013; Hood et al., 2018). Our previous study indicated that ER stress inhibition significantly protected against neuronal apoptosis after spinal cord injury (He et al., 2017), but the role of ER stress in TBI is still unclear. An increasing number of studies suggested that vitamin B_12_ regulated ER homeostasis (Sukocheva et al., 2001; Ghemrawi et al., 2013). Furthermore, it was reported that vitamin B_12_ deficiency activated ER stress pathways by increasing the phosphorylation of PERK and IRE1α and the expression of ATF6 (Ghemrawi et al., 2013). Given our previous work on ER stress and its participation in nerve disease, we hypothesized that vitamin B_12_ could promote nerve regeneration after TBI by regulating ER stress.

In this study, we explored the effect of vitamin B_12_ on nerve regeneration after TBI both *in vivo* and *in vitro*. Meanwhile, we investigated the role of ER stress during the vitamin B_12_ treatment for TBI by inspecting changes in neuronal apoptosis, microtubule, myelin regeneration, and axonal growth.

## Materials and Methods

### Animals

Adult C57BL/6 male mice aged 6–8 weeks and weighing 20–26 g were purchased from the Animal Center of the Chinese Academy of Sciences (Shanghai, China). Animals were housed under a 12-h light/dark cycle at 21–23°C and provided access to food and water *ad libitum*. The care and use of all animals were approved by the Ethics Committee of Wenzhou Medical University and conformed to the guidelines set forth by the Chinese National Institutes of Health. Mice were allocated to the following four groups using a random number table: the sham animal group, which received only anesthesia and a craniotomy: the TBI group; and two vitamin B_12_ treatment groups (doses of 0.5 mg/kg/day and 1.5 mg/kg/day) that received vitamin B_12_ intraperitoneally following surgery.

### Cell Culture and OGD/Re-oxygenation Model

PC12 cells were purchased from the Cell Bank of the Type Culture Collection of the Chinese Academy of Sciences, Shanghai Institute of Cell Biology, Chinese Academy of Sciences. The PC12 cells were highly differentiated and adhered. Cells were cultured with RPMI 1640 supplemented with 10% FBS, 100 U/mL penicillin and 100 μg/mL streptomycin, and then incubated in a humidified atmosphere containing 5% CO_2_ at 37°C. For oxygen-glucose deprivation (OGD), cells were incubated in an anaerobic chamber for 6 h at an oxygen level that remained below 0.5% with normal growth medium or FBS-free medium, and then cells were incubated for another 12 h under normal culture conditions. Vitamin B_12_ (200 μM) pretreatment was administered for 2 h before OGD. To further estimate the effect of ER stress activation during OGD, cells were pretreated with 4-phenylbutyric acid (4-PBA) (1 mM) for 1 h. All experiments were performed at least three times.

### Surgical Procedures

TBI procedures were performed in male mice by controlled cortical impact (CCI). Briefly, all male mice were anesthetized with isoflurane via brain stereotaxis, and an incision was made in the middle of the scalp to expose the skull. After removing the bone flap, CCI was performed with a controlled impactor device at a speed of 4 m/s, a depth of 1 mm and a 150-ms impact duration (Impact One^TM^ Stereotaxic Impactor, Leica, Milan, Italy). Postoperatively, the incision was sutured and treated with a local antibiotic application (cefazolin sodium salt). The mice were placed on a heating pad at 37°C to recover from anesthesia. All experiments were conducted with all efforts to reduce the number of animals used and their suffering as much as possible.

### Drug Administration

Vitamin B_12_ (V2876, Sigma) and 4-PBA (P21005, Sigma) were used as drugs in this study. The vitaminB_12_ was dissolved in normal saline as 150 mg/ml stock. The mice were treated with vitamin B_12_ in 0.5 mg/kg or 1.5 mg/kg by intraperitoneal injection from 1 day after injury for 2 consecutive weeks. The 4-PBA (100 mg/kg) was administered before vitamin B_12_ treatment via intraperitoneal injection. Every drug was filtered by 0.2 μm microfiltration membrane before injection.

### Brain Water Content

Mice were decapitated under deep anesthesia and perfusion at 24 h after TBI induction. The olfactory bulbs and brain stems were removed from their brains. Then brains were divided into two segments: right hemispheres and left hemispheres. Each part of the brain was weighed immediately to determine the wet weight. The samples were dried at 72°C for 72 h to obtain dry weights. Brain water contents were calculated as follows: [(wet weight - dry weight)/wet weight] × 100%.

### Garcia Neurobehavioral Score

According to Garcia neurobehavioral score, the mice in each group were scored on day 1, day 7, and day 14 after TBI. The recovery of nerve function after TBI was observed by double-blind method. Briefly, (1) spontaneous activity (0 ≤ 3 points). (2) symmetrical movement of limbs (0 ≤ 3 points). (3) forelimb extension (0 ≤ 3 points). (4) climbing (0–3 points). (5) body trunk reaction (0 ≤ 3 points). (6) tentacles reaction (0 ≤ 3 points). (7) lateral rotation reaction (0 ≤ 3 points).

### Tissue Preparation

Animals were anesthetized with isoflurane at specific time points following TBI. For immunofluorescence staining, brain tissues were dissected out, post-fixed by 4% paraformaldehyde (PFA) for 12 h, embedded in paraffin, cut into 5 mm sections and mounted on slides for subsequent staining. For western blot, a brain segment was dissected and stored at -80°C immediately. Animal tissues were lysed with RIPA lysis buffer (50 mM Tris, 150 mM NaCl, 1% Triton X-100,1% sodium deoxycholate, 0.1% SDS, 5 mM sodium orthovanadate, 5 mM sodium fluoride, 1 mM and EDTA, pH 7.4) supplemented with 10 μl/ml protease inhibitor cocktail (GE Healthcare Biosciences, Pittsburgh, PA, United States). The samples were homogenized using mechanical disruption and ultrasound cell breaker (30 s/times in three times). The tissue lysates were incubating at 4°C for 15 min. The supernatants were collected after centrifuging at 12,000 rpm at 4°C for 15 min.

### Animal Experiment

Luxol fast blue (LFB) staining: The sections were deparaffinized, rinsed in dimethylbenzene I, dimethylbenzene II, absolute ethyl alcohol I, absolute ethyl alcohol II, 95% ethanol, 90% ethanol, 80% ethanol, 70% ethanol, and distilled water and then incubated in an LFB solution (0.01% in 95% ethanol) overnight at 60°C. Gray and white matter tissues were differentiated by immersing the slides in 0.05% lithium carbonate solution for 5 – 10 s, followed by 2 changes of 70% ethanol for 1 min each, and then rinsed in distilled water. The sections were observed under a microscope to confirm proper differentiation. Differentiation steps were repeated until a sharp contrast was achieved between blue-stained white matter and colorless gray matter.

Nissl staining: We dewaxed the paraffin sections and stained them with 5% toluidine blue at room temperature for 10 min. Sections were soaked in 95% ethanol for 2 min, dimethylbenzene for 3 min and sealed with neutral balsam. The Nissl-stained slides were observed using a Nikon microscope.

H&E staining: An H&E staining kit was purchased from Beyotime Company (Jiangsu, China), and the experimental procedure was performed according to the kit instructions.

Immunohistochemistry: Paraffin sections were deparaffinized by dimethylbenzene and then rehydrated in an alcohol gradient. After incubated in 3% oxydol for 15 min, antigen retrieval was performed in citric acid by a pressure cooker, and then the slides were cooled for 2 h. BSA at 5% was used for pre-incubation at 37°C for 30 min, and the sides were incubated with the primary antibodies at 4°C for 24 h. The following primary antibody was used: MBP (1:1000, Cell Signaling Technologies, America). In the next step, glass slides were incubated with goat anti-rabbit IgG (H+L) HRP secondary polyclonal antibody (1:1000, Yeason) at 37°C for 1 h and then with DAB for approximately 5 min. Stained sections were photographed with a fluorescence microscope (Nikon, Tokyo, Japan).

### Western Blot

Proteins from tissues and cells were quantified with BCA reagents. 40 μg proteins were separated on a 12% gel and transferred onto a polyvinylidene fluoride (PVDF) membrane (Bio-Rad, Hercules, CA, United States). The membranes were blocked for 120 min with 5% (w/v) milk dissolved in 0.1% Tween-20 in TBS at room temperature, and then were incubated overnight at 4°C with the following primary antibodies: Caspase12 (1:1000, Abcam, United States), IRE1α (1:1000, Abcam, United States), GRP78 (1:1000, Abcam, United States), XBP-1 (1:1000, Abcam, United States), CHOP (1:1000, Cell Signaling Technologies, United States), Ace-tubulin (1:2000, Cell Signaling Technologies, United States), Tau (1:1000, Abcam, United States), MAP2 (1:2000, Cell Signaling Technologies, United States), myelin basic protein (MBP) (1:1000,Cell Signaling Technologies, United States), and GAPDH (1:10000, Bio-world, United States). Subsequently, the membranes were washed thrice with TBST and the membranes were incubated with horseradish-peroxidase conjugated secondary antibodies rabbit/mouse polyclonal antibody (1:1000, Yeason) for 60 min. A ChemiDoc^TM^ XRS imaging system (Bio-Rad, United States) was used to visualize the signals. Quantity One was used to analyze the relative band densities, and the band densities of target proteins were normalized to that of GAPDH. All experiments were repeated at least in triplicate.

### Immunofluorescence Staining

Cells were fixed with 4% PFA for 30 min at 37°C. The prepared tissue sections and cells were blocked with 5% BSA for 30 min and then incubated at 4°C overnight with the following primary antibodies: MAP-2 (1:500, Abcam, United States), Ace-tubulin (1:500, Cell Signaling Technologies, United States), Tyr-tubulin (1:500, Sigma Aldrich, United States), GRP78 (1:1000, Abcam, United States), and MBP (1:500, Cell Signaling Technologies, United States). Next, the sections and cells were incubated with an Alexa Fluor 594/647 donkey anti-mouse/rabbit secondary antibody (1:1000, Abcam, United States) for 1 h at 37°C. Nucleis were stained with DAPI. The samples were imaged under a Nikon ECLIPSE Ti microscope (Nikon, A1 PLUS, Tokyo, Japan). 10 images were captured from each sample in the cortex randomly. The quantitative of immunofluorescence was performed by Image J. All experiments were repeated at least in triplicate.

### TUNEL Staining

The TUNEL analysis kit (40307ES20, Yeason) was used for detecting the level of DNA damage. The brain sections were deparaffinized and rehydrated by difference concentrated-ethanol. After washing with PBS, the sections were treated with 10.2 mM sodium citrate buffer. The sections were stained with Alexa Fluor 488-12-dUTP Labeling Mix. The sections were counter staining with DAPI after washing with PBS. The images were captured by the fluorescence microscope (Olympus Inc., Tokyo, Japan). The TUNEL positive cells in six random fields of each section were counted for analysis.

### Statistical Analysis

Statistically analyzed data were presented as the mean ± standard error of the mean (SEM). Student’s *t*-test was used to determine statistical significance between two groups. For comparison of three or more groups, one-way analysis of variance (ANOVA) followed by Tukey’s *post hoc* test was used to analyze the results. The difference was considered statistically significant when the *P*-value was <0.05.

## Results

### Vitamin B12 Decreased En-Cephaledema and Motor Neuron Loss After TBI

**FIGURE 1:**
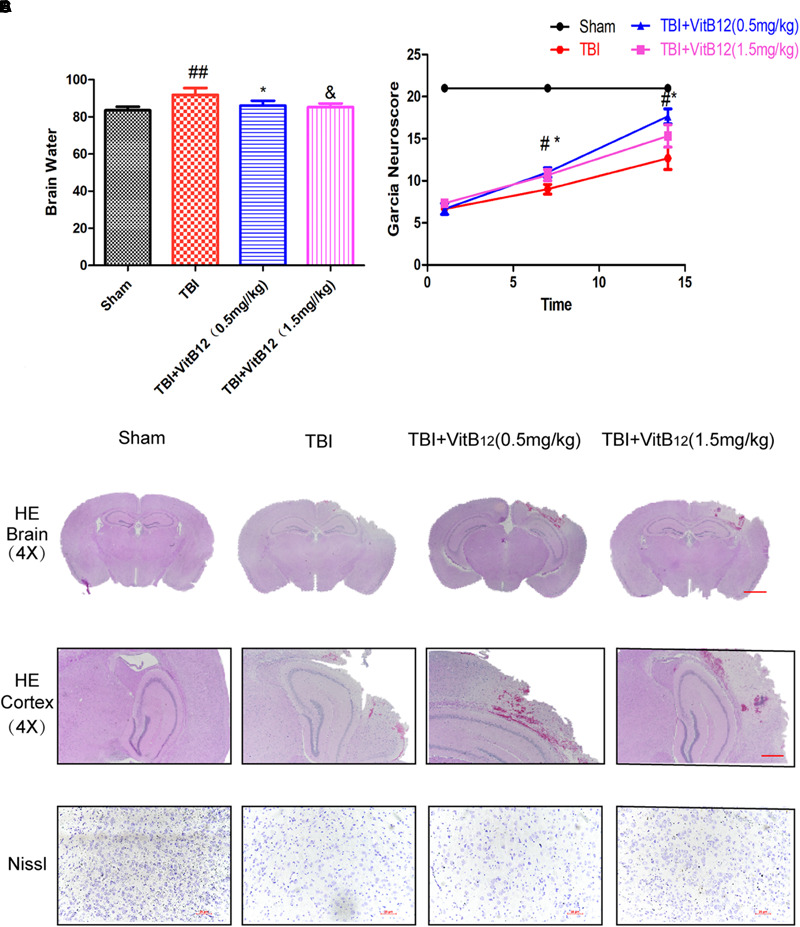
Vitamin B12 treatment reserves tissue structure damage and protects functional recovery after TBI. **(A)** Quantification of brain water content in the ipsilateral brain cortex at 1 day after TBI. ^##^*P* < 0.01 vs. the sham group. ^∗^*P* < 0.05 and ^&^*P* < 0.05 vs. the TBI group values represent the mean ± SEM, *n* = 6. **(B)** Garcia test evaluation at 7 days, 14 days after TBI. ^#^*P* < 0.05 vs. the sham group. ^∗^*P* < 0.05 vs. the TBI group values represent the mean ± SEM, *n* = 5. **(C–D)** Representative images of H&E and Nissl staining in the cortex at 14 days post-TBI.

To evaluate the therapeutic role of vitamin B12 in the treatment of TBI, vitamin B12 was administered intraperitoneally immediately following TBI. Brain water content was detected at 24 h after TBI. The brain water content of the TBI group was obviously increased relative to the sham group (91.91 ± 1.488 vs. 83.53 ± 0.7815, Figure 1A, Mean ± SEM, *n* = 6), and the brain water contents of the vitamin B12-treated TBI groups were reduced to differing degrees relative to the TBI group (86.11 ± 1.070 in 0.5 mg/kg vitamin B12 dose group and 85.31 ± 0.7846 in 1.5 mg/kg vitamin B12 dose group vs. 91.91 ± 1.488 in TBI group, Mean ± SEM, *n* = 6). These results indicated that vitamin B12 significantly alleviated ipsilateral brain oedema after TBI injury.

Motor function recovery was estimated for 14 days after injury using the 21-point Garcia test. As shown in Figure 1B, the sham-operated group had an average score of 21, representing a normal motor function. The TBI+vitamin B12 group showed better functional recovery after 7 days. Additionally, H&E and Nissl staining were performed to assess the histological morphology in each group. As shown in Figure 1C, there was obvious severe cerebral cortex tissue loss in the TBI group relative to the sham group. Consistent with the Garcia scores, the vitamin B12-treated groups showed less tissue damage and neuronal apoptosis (Figure 1D), indicating that vitamin B12 reduced tissue damage, protected neurons in the cortex and ameliorated the pathological morphology of the lesion area after TBI in mice.

### Vitamin B12 Alleviated Caspase12-Dependent Neuronal Apoptosis

**FIGURE 2:**
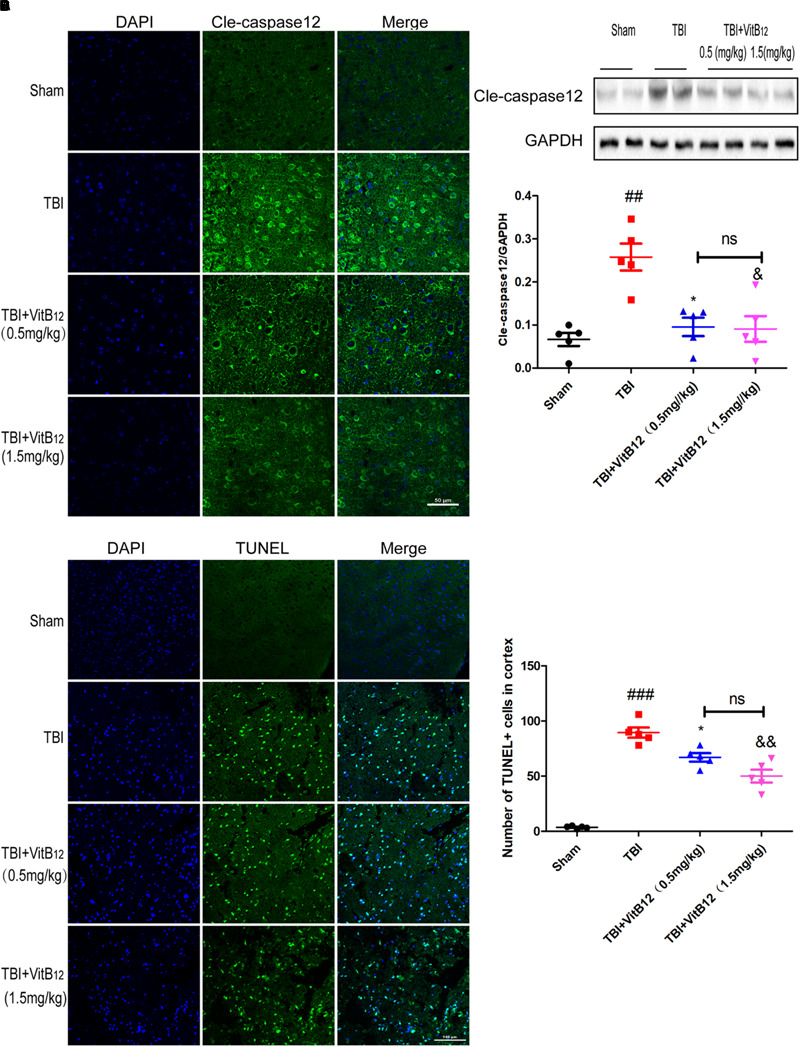
Vitamin B12 protects neurons from apoptosis. **(A)** Immunofluorescence staining of cleaved caspase-12 (green) in the cortex at 7 days post-injury. Scale bar = 50 μm. **(B,C)** The expression and quantification of cleaved caspase-12 proteins in the cortex in the 7 days post-injury. ^##^*P* < 0.01 vs. the sham group. ^∗^*P* < 0.05 and ^&^*P* < 0.05 vs. the TBI group values represent the mean ± SEM, *n* = 5. **(D,E)** Representation and quantification of immunofluorescence staining of TUNEL (green) in the cortex at 7 days post-injury. ^##^*P* < 0.01 vs. the sham group. ^∗^*P* < 0.05 and ^&^*P* < 0.05 vs. the TBI group values represent the mean ± SEM, *n* = 5. Scale bar = 100 μm.

To determine whether vitamin B12 treatment could decrease apoptosis in brain tissues, immunofluorescence staining was performed after TBI. As shown in Figure 2A, vitamin B12 treatment obviously reduced the amount of cleaved-caspase12 positive neurons relative to the TBI group. Meanwhile, western blot results indicated that the vitamin B12-treated groups showed reduced levels of cleaved-caspase12 expression relative to their untreated counterparts in Figure 2B,C. Moreover, TUNEL staining was performed on 7 days after injury to further verify the anti-apoptotic effect of vitamin B12. As shown in Figure 2D,E, significantly increased number of apoptotic cells was found in the TBI group relative to the sham group. In comparison, vitamin B12 treatment greatly reversed TBI-induced apoptosis. Therefore, these findings demonstrated that vitamin B_12_ attenuated TBI-induced neuronal cell apoptosis.

### Vitamin B12 Inhibited ER Stress Signaling Pathway

**FIGURE 3:**
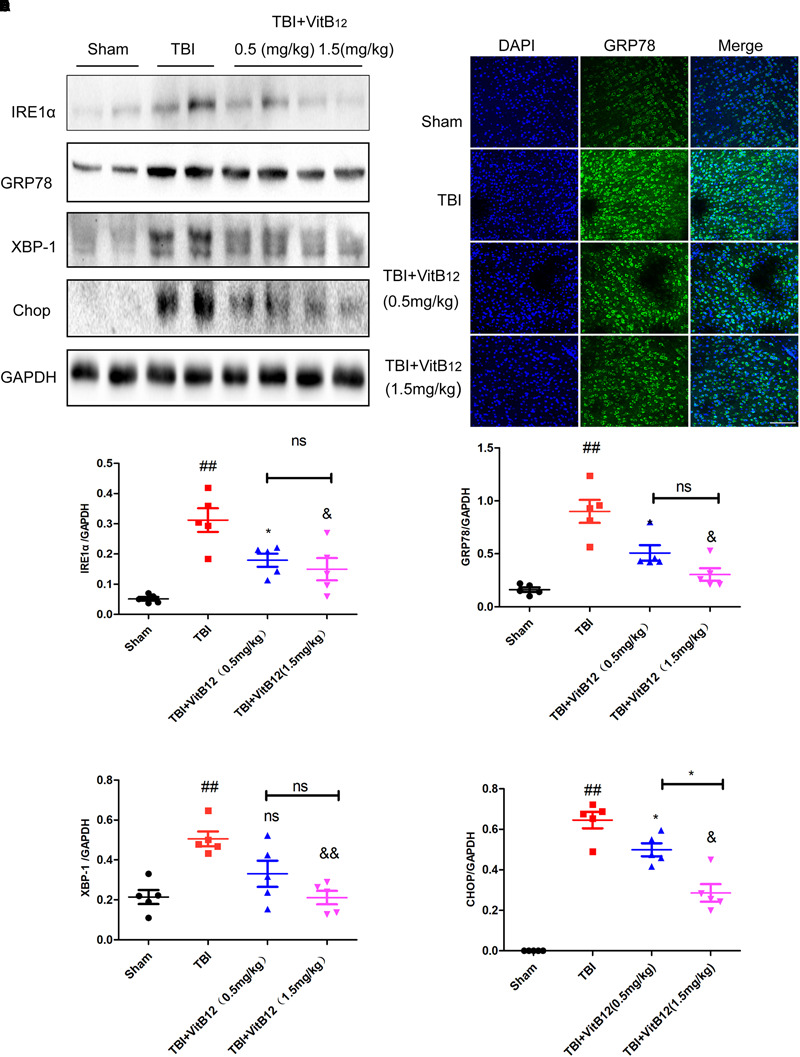
Vitamin B12 reduces the level of ER stress. **(A)** Representative western blots of the expression of IRE1α, GRP78, XBP-1, Chop. **(B)** Immunofluorescence staining of GRP78 (green) in the cortex at 7 days post-injury. Scale bar = 100 μm. **(C–F)** Quantification from **(A)** ^##^*P* < 0.01 vs. the sham group. ^∗^*P* < 0.05, ^&^*P* < 0.05, and ^&&^*P* < 0.01 vs. the TBI group. ^∗^*P* < 0.05 vs. the indicated group. Values represent the mean ± SEM, *n* = 5.

Under chronic ER stress, the associated apoptosis may contribute to pathophysiological processes involved in a number of prevalent diseases, including neurodegenerative diseases, diabetes, atherosclerosis and renal disease (Tabas and Ron, 2011). Hu et al. (Wu et al., 2018) reported that the ER-localized E3 ligase RNF183 triggered apoptosis in response to prolonged ER stress. Meanwhile, our previous study indicated that ER stress signaling induced neuronal apoptosis in spinal cord injury (He et al., 2017). To clarify the relationship between vitamin B12 and the regulation of ER stress, we detected ER stress signaling pathway proteins and the downstream apoptosis-related proteins by western blot and immunofluorescence staining. As shown in Figure 3A–F, the levels of ER stress-related proteins (GRP78, IRE1α, XBP-1, and CHOP) were significantly increased at 3 days and decreased after vitamin B12 treatment. Meanwhile, immunofluorescence staining revealed that the number of GRP78 positive cells was significantly increased in the TBI group, and vitamin B12 reversed this trend to a differing degree (Figure 3B). These findings suggested that vitamin B12 alleviated the level of ER stress.

### Vitamin B12 Inhibited Microtubule Damage Through ER Stress After TBI

**FIGURE 4:**
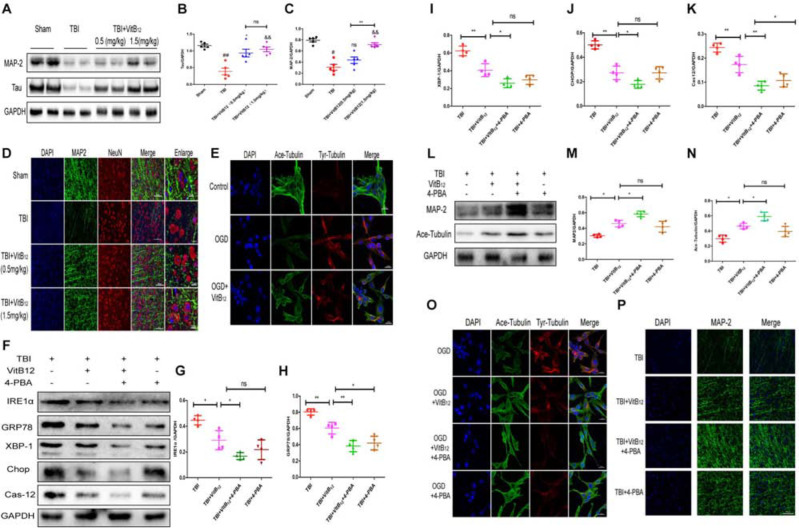
Vitamin B12 inhibits microtubule damage through ER stress after TBI. **(A)** Representative western blots of the expression of MAP-2, Tau. **(B,C)** Quantification from **(A)** ^##^*P* < 0.01, ^#^*P* < 0.05 vs. the sham group. ^∗^*P* < 0.05, ^&&^*P* < 0.01 vs. the TBI group. ^∗∗^*P* < 0.01 vs. the indicated group. Values represent the mean ± SEM, *n* = 5. **(D)** Representative fluorescent images depicting MAP-2 (green) with NeuN (red) in the cortex after TBI. Scale bar = 50 μm, scale bar = 10 μm. **(E)** Representative fluorescent images depicting Ace-tubulin (green) with Tyr-tubulin (red) in the PC 12 cells. Scale bar = 20 μm. **(F)** Representative western blots of the expression of IRE1α, GRP78, XBP-1, Chop, Cleaved-cas12. **(G–K)** Quantification from **(F)** ^∗^*P* < 0.05, ^∗∗^*P* < 0.01, and ^∗∗∗^*P* < 0.001 vs. the indicated group. Values represent the mean ± SEM, *n* = 4. **(L)** Representative western blots of the expression of MAP – 2, Ace-tubulin. **(M,N)** Quantification from **(L)**. ^∗^*P* < 0.05 vs. the indicated group. Values represent the mean ± SEM, *n* = 4. **(O)** Representative fluorescent images depicting Ace-tubulin (green) with Tyr-tubulin (red) in the PC 12 cells. Scale bar = 20 μm. **(P)**. Representative fluorescent images depicting MAP-2 (green) *in vivo*. Scale bar = 50 μm.

Numerous studies have indicated that vitamin B12 has neuroprotective effects on TBI (Sun et al., 2012; Chen et al., 2015). We hypothesized that vitamin B12 may play a neuroprotective role by maintaining microtubule stability. We detected the expressions of MAP-2 and Tau, promote axon microtubulin bundling and dynamics and stabilize microtubule. As shown in Figure 4A–C, the expressions of MAP-2 and Tau decreased after injury, and the Tau expression was significantly higher in the vitamin B12-treated group relative to the TBI group after 7 days. Interestingly, only the 1.5 mg/kg dose of vitamin B12 reversed the loss of MAP-2. Then we examined MAP-2 expression by immunofluorescence. The results revealed that the MAP-2-positive neurons in the TBI group were significantly disorganized 7 days after injury. However, the vitamin B12-treated groups showed good neurological morphology, which was similar to the morphology observed in the sham group relative to the TBI group (Figure 4D). In addition, this result was further confirmed by immunofluorescence in PC12 cells with OGD, as shown in Figure 4E. The Ace-tubulin and Tyr-tubulin proteins were detected to examine stable microtubules and dynamic microtubules, respectively. The results showed that the Ace-tubulin/Tyr-tubulin ratio after vitamin B12 treatment was increased relative to the OGD treatment group. Taken together, vitamin B12 exerted microtubule stabilizing effect.

We next explored the relationship between vitamin B12 protection and ER stress by using 4-PBA, which is the most commonly used ER stress inhibitor. As shown in Figure 4F–K, ER stress-induced apoptosis signaling pathway levels were substantially down-regulated in the vitamin B12 and 4-PBA co-treatment group relative to the vitamin B12 group. Meanwhile, microtubule stabilization protein expressions (MAP-2 and Ace-tubulin) were significantly increased in the co-treatment group relative to the vitamin B12 group, which indicated that vitamin B12-mediated protection was most likely achieved by inhibiting ER stress (Figure 4L–N). Next, we used immunofluorescence to detect Ace-tubulin and Tyr-tubulin in PC12 cells *in vitro* and MAP-2 *in vivo*. The results showed that the Ace-tubulin/Try-tubulin ratio in the vitamin B12+4-PBA group was increased relative to the vitamin B12 group, which was consistent with MAP-2 in mice (Figure 4O,P). Taken together, these results suggested that vitamin B12 was able to stabilize microtubules by reducing ER stress level.

### Vitamin B12 Promoted Myelin Regeneration by ER Stress After TBI

**FIGURE 5:**
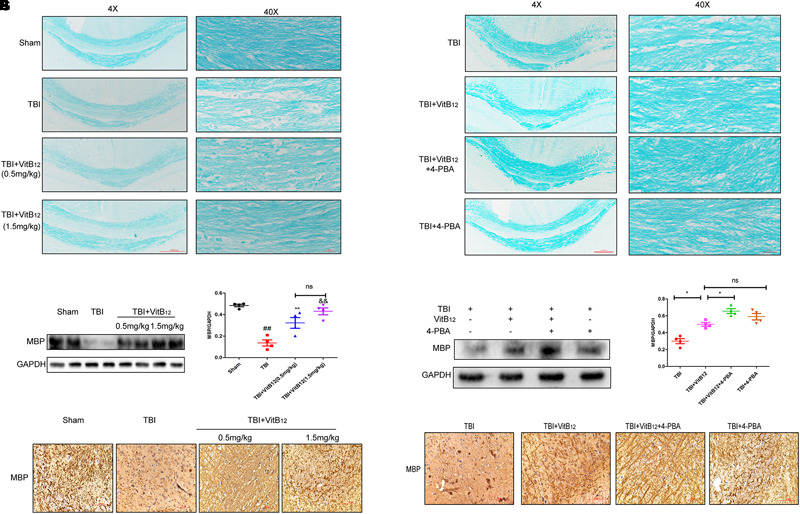
Vitamin B12 protects against myelin damage after TBI through ER Stress. **(A)** Representative images of white matter with LFB staining images of the myelin sheath at 14 days. Scale bar = 1000 μm, Scale bar = 250 μm. **(B,C)** Protein expressions and quantification data of MBP in each group. ^##^*P* < 0.01 vs. the sham group, ^∗∗^*P* < 0.01 and ^&&^*P* < 0.01 vs. the TBI group. Values represent the mean ± SEM, *n* = 4. **(D)** Immuno-histochemisty staining of MBP in each group. **(E)** Representative images of white matter with LFB staining images of the myelin sheath at 14 days. Scale bar = 1000 μm, Scale bar = 250 μm. **(F,G)** Protein expressions and quantification data of MBP in each group. ^∗^*P* < 0.05 vs. the indicated group values represent the mean ± SEM, *n* = 4. **(H)** Immuno-histochemisty staining of MBP in each group.

Myelin regeneration is a key factor in sensory and motor function recovery after brain injury (Cantuti-Castelvetri et al., 2018). We explored the effect of vitamin B12 on re-myelination by LFB staining. Histopathology with LFB staining revealed that the TBI group showed white matter lesions in the corpus callosum relative to the sham group (Figure 5A). Vacuolar changes and microtubule loss from the myelin sheath were also observed in the TBI group. The vitamin B12 treatments reduced the degree of myelin sheath destruction, and 1.5 mg/kg dose of vitamin B12 showed slightly better effects than the 0.5 mg/kg dose. Next, MBP, which is constituents of the myelin sheath was detected by western blot and immunohistochemistry. As shown in Figure 5B–D, MBP protein expression was significantly decreased after TBI, but increased by vitamin B12; there was no statistical significance between the two groups receiving different vitamin B12 concentrations. Taken together, these results revealed that vitamin B12 promoted myelin regeneration after TBI. We next explored whether ER stress was involved in the protective effect of vitamin B12 on remyelination. LFB staining revealed that the myelin sheath in the corpus callosum was clear, and the microtubules in the axons were tightly arranged in the vitamin B12+4-PBA group relative to vitamin B12 treatment group (Figure 5E). Moreover, the expression of MBP was consistent with the LFB staining. As shown in Figure 5F,G, the expression of MBP was significantly increased in the co-treatment group relative to the vitamin B12 treatment groups. Meanwhile, immunohistochemistry results showed that the vitamin B12+4-PBA group presented tighter and more continuous MBP positive myelin relative to that in the vitamin B12 groups (Figure 5H). All these results indicated that the vitamin B12 enhanced re-myelination by inhibiting ER Stress.

## Discussion

Increased understanding of neuroprotection in TBI has helped to establish the concept of promoting nerve cell function recovery to potentially improve functional recovery (Wieloch and Nikolich, 2006; Loane and Faden, 2010). Numerous studies have reported that microtubule stabilization and remyelination play pivotal roles in several CNS diseases (Sengottuvel and Fischer, 2011; Abu-Rub and Miller, 2018; Li et al., 2018). There is a consistent evidence that vitamin B12 promotes the synthesis of neurotrophic factors, which in turn support neurite outgrowth and survival (Okada et al., 2010). However, whether vitamin B12 can alleviate the damage caused by brain trauma is still unclear. To assess this possibility, vitamin B12 was administered intraperitoneally immediately following TBI. And the results showed that vitamin B12 was able to promote nerve repair after TBI.

The ER is the main organelle responsible for protein folding, lipid biosynthesis and Ca^2+^ storage (Tsai and Weissman, 2010). Increased ER stress may be a self-protective signal transcription pathway after mild injury (Xie et al., 2016), In contrast, excessive ER stress triggers extensive neuronal death via CHOP activation. CHOP is the downstream of ER stress-induced apoptosis. The cytoplasmic calcium-activated calpain cleaves and activates caspase-12 in response to ER-released calcium (Hood et al., 2018; Tan et al., 2018). It has been confirmed that, ER stress is also involved in TBI (Hood et al., 2018). Vitamin B12 has been currently used to treat peripheral nerve damage in the clinic (Hobbenaghi et al., 2013). In this study, we found that the ER stress was involved in the process of vitamin B12 treating TBI (Figure 3). As showed in Figure 3A, IRE1α, GRP78, XBP-1 and CHOP were increased significantly after TBI injury, and reversed following the 3-day treatment of vitamin B12_._ Meanwhile, vitamin B12 alleviated ER stress-induced apoptosis. Taken together, ER stress plays an important role in neuronal apoptosis induction after TBI, and vitamin B12 inhibits neuronal death by down-regulating ER stress.

A growing number of studies has reported that microtubule stabilization might play a pivotal role in axon regeneration in several CNS diseases (O’Donovan, 2016; Abu-Rub and Miller, 2018; Chuckowree et al., 2018). In an injured brain, microtubules are vulnerable to misalignment and dissolution in neurons. And microtubules also implicate in the injury-induced glial responses and adaptive neuroplasticity in the aftermath of injury (Chuckowree et al., 2018). Tau protein is involved in regulating axonal microtubule assembly and disassembly. It has been reported that the plasma phospho-tau levels and phospho tau/total tau ratio during the acute phase and chronic TBI were superior to total/tau levels as discriminating indices for the severity and status of neurotrauma patients from healthy controls (Rubenstein et al., 2017). This study also demonstrated that the vitamin B12 could maintain the stability of the microtubule after TBI. As shown in Figure 4, vitamin B12 exerted microtubule-stabilizing effect *in vitro* and *in vivo*. Meanwhile, the high dose (1.5 mg/kg) of vitamin B12 treatment showed better effectiveness than the 0.5 mg/kg dose in TBI mice. In addition, the combination therapy with the ER stress inhibitor 4-PBA partially strengthened the neuro-protective effect of vitamin B12. However, whether vitamin B12 directly inhibits the ER stress signaling pathway requires further investigation.

CNS injury-induced growth cone collapse and retraction of the axonal cytoskeleton are closely related to growth inhibitory molecules associated with myelin (Stassart et al., 2018). The myelinating cells surrounding axons not only accelerate the propagation of electrical impulses, but also provide metabolic support for axons and refine neural circuits (Figlia et al., 2017). Myelin has a significant role in the progression of white matter pathology after TBI and in the potential for plasticity and subsequent recovery (Armstrong et al., 2016). Besides, myelin is an active form of vitamin B12, and the MeCbl plays an essential role in the synthesis and maintenance of myelin (Gröber et al., 2013). Vitamin B12 accelerates Schwann cells differentiation by suppressing Erk1/2 activities (Nishimoto et al., 2015). In addition, vitamin B12 has been reported to promote the remyelination in focal demyelination rat (Nishimoto et al., 2015). Thus, we evaluated the effect of vitamin B12 on remyelination after TBI. These data suggested that vitamin B12 increased the level of MBP, which plays vital roles in the myelination process and the appropriate formation of myelin thickness and compactness. Meanwhile, LFB staining showed that vitamin B12 restored myelin by reducing the vacuolar changes in the myelin sheath after TBI. We also used the ER stress inhibitor 4-PBA to assess the role of ER stress in remyelination. The results showed that ER stress was involved in the treatment of TBI induced myelin damage by vitamin B12. Therefore, we can conclude that the vitamin B12 enhance the survival of the nerve cell in the TBI mouse by inhibiting ER stress. In our further study, we will investigate the effects of vitamin B12 on glial cells and the relationship between membrane-associated proteins and autophagy signals, ultimately meeting the goal of deeper understand of the treatment mechanism of vitamin B12 in TBI.

## Ethics Statement

The care and use of all animals were approved by the Ethics Committee of Wenzhou Medical University and conformed to the guidelines set forth by the Chinese National Institutes of Health.

## Author Contributions

AH and DC conceived and designed the experiments. FW, KX, LL, MZ, and CT performed the experiments, analyzed data and wrote or revised the manuscript. KZ, LX, HT, YH, and YX provided assistance with experiments. HZ was responsible for experiment supplementation and manuscript modification. All the above authors discussed the results and approved the manuscript submission.

## Conflict of Interest Statement

The authors declare that the research was conducted in the absence of any commercial or financial relationships that could be construed as a potential conflict of interest.
