# Corrigendum: Vitamin B12 Enhances Nerve Repair and Improves Functional Recovery After Traumatic Brain Injury by Inhibiting ER Stress-Induced Neuron Injury

**DOI:** 10.3389/fphar.2021.598335

**Published:** 2021-04-12

**Authors:** Fangfang Wu, Ke Xu, Lei Liu, Kairui Zhang, Leilei Xia, Man Zhang, Chenhuai Teng, Heyan Tong, Yifang He, Yujie Xue, Hongyu Zhang, Daqing Chen, Aiping Hu

**Affiliations:** ^1^ Department of Emergency, The Second Affiliated Hospital and Yuying Children’s Hospital, Wenzhou Medical University, Wenzhou, China; ^2^ School of Pharmaceutical Sciences, Wenzhou Medical University, Wenzhou, China; ^3^ Institute of Life Sciences, Wenzhou University, Wenzhou, China; ^4^ Department of Emergency, Wenzhou People’s Hospital, The Third Clinical Institute Affiliated to Wenzhou Medical University, Wenzhou, China

**Keywords:** vitamin B12, traumatic brain injury, endoplasmic reticulum stress, microtubule, myelin

In the original article, there was a mistake in **Figure 4P** as published. The corrected Figure 4 appear below.

The authors apologize for this error and state that this does not change the scientific conclusions of the article in any way.

**FIGURE 4:**
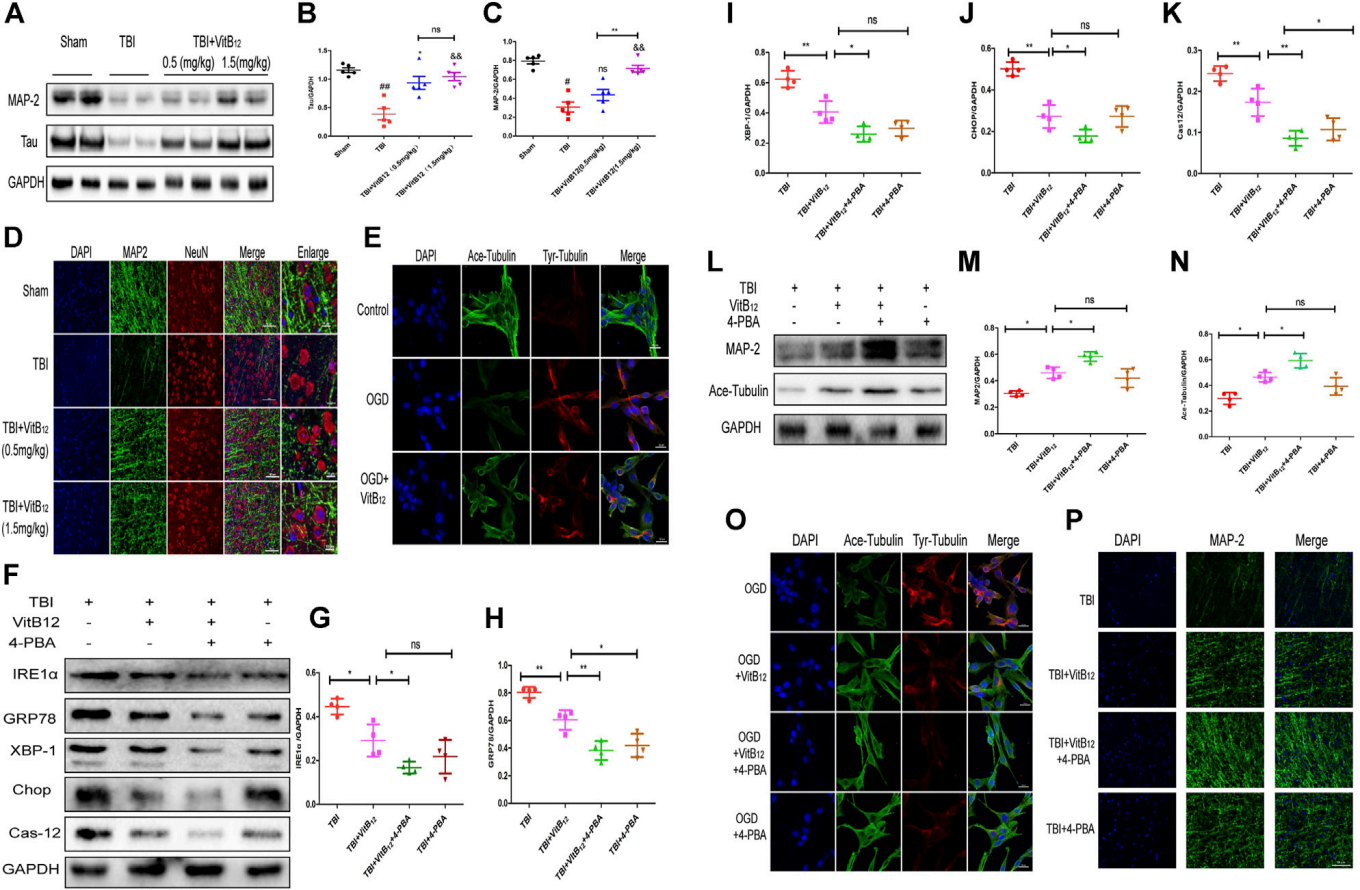
Vitamin B12 inhibits microtubule damage through ER stress after TBI. **(A)** Representative western blots of the expression of MAP-2, Tau. **(B, C)** Quantification from **(A)**.##*p* < 0.01, #*p* < 0.05 vs. the sham group. *p < 0.05, && *p* < 0.01 vs. the TBI group. **p < 0.01 vs. the indicated group. Values represent the mean ± SEM, n = 5. **(D)** Representative fluorescent images depicting MAP-2 (green) with NeuN (red) in the cortex after TBI. Scale bar = 50 μm, scale bar = 10 µm. **(E)** Representative fluorescent images depicting Ace-tubulin(green) with Tyr-tubulin(red) in the PC 12 cells. Scale bar = 20 µm. **(F)** Representative western blots of the expression of IRE1α, GRP78, XBP-1, Chop, Cleaved-cas12. **(G–K)** Quantification from (F)*p < 0.05, **p < 0.01 and ***p < 0.001 vs. the indicated group. Values represent the mean ± SEM, n = 4. **(L)** Representative western blots of the expression of MAP-2, Ace-tubulin. **(M–N)** Quantification from **(L)**.*p < 0.05 vs. the indicated group. Values represent the mean ± SEM, n = 4. **(O)** Representative fluorescent images depicting Ace-tubulin(green) with Tyr-tubulin(red) in the PC 12 cells. Scale bar = 20 µm. **(P)**. Representative fluorescent images depicting MAP-2 (green) *in vivo*. Scale bar = 50 µm.

